# Universal detection of curved rice panicles in complex environments using aerial images and improved YOLOv4 model

**DOI:** 10.3389/fpls.2022.1021398

**Published:** 2022-11-07

**Authors:** Boteng Sun, Wei Zhou, Shilin Zhu, Song Huang, Xun Yu, Zhenyuan Wu, Xiaolong Lei, Dameng Yin, Haixiao Xia, Yong Chen, Fei Deng, Youfeng Tao, Hong Cheng, Xiuliang Jin, Wanjun Ren

**Affiliations:** ^1^ State Key Laboratory of Crop Gene Exploration and Utilization in Southwest China, Key Laboratory of Crop Ecophysiology and Farming System in Southwest China, Ministry of Agriculture, Sichuan Agricultural University, Chengdu, Sichuan, China; ^2^ Key Laboratory of Crop Physiology and Ecology, Ministry of Agriculture, Institute of Crop Sciences, Chinese Academy of Agricultural Sciences, Beijing, China

**Keywords:** curved rice panicle, panicle recognition model, YOLOv4, MobileNetv2, UAV, convolutional block attention module

## Abstract

Accurate and rapid identification of the effective number of panicles per unit area is crucial for the assessment of rice yield. As part of agricultural development, manual observation of effective panicles in the paddy field is being replaced by unmanned aerial vehicle (UAV) imaging combined with target detection modeling. However, UAV images of panicles of curved hybrid Indica rice in complex field environments are characterized by overlapping, blocking, and dense distribution, imposing challenges on rice panicle detection models. This paper proposes a universal curved panicle detection method by combining UAV images of different types of hybrid Indica rice panicles (leaf-above-spike, spike-above-leaf, and middle type) from four ecological sites using an improved You Only Look Once version 4 (YOLOv4) model. MobileNetv2 is used as the backbone feature extraction network based on a lightweight model in addition to a focal loss and convolutional block attention module for improved detection of curved rice panicles of different varieties. Moreover, soft non-maximum suppression is used to address rice panicle occlusion in the dataset. This model yields a single image detection rate of 44.46 FPS, and mean average precision, recall, and F1 values of 90.32%, 82.36%, and 0.89%, respectively. This represents an increase of 6.2%, 0.12%, and 16.24% from those of the original YOLOv4 model, respectively. The model exhibits superior performance in identifying different strain types in mixed and independent datasets, indicating its feasibility as a general model for detection of different types of rice panicles in the heading stage.

## 1 Introduction

There is a great need to improve rice yield as rice (indica hybrid rice (*Oryza Satiua* L.)) is the staple food of 60% of China’s population (Zhou et al., 2016). Effective panicles per unit area is a key determinant of rice yield and its accurate detection can guide the development of cultivation techniques for high-yield and high quality rice (Slafer et al., 2014). Currently, manual selection statistics are used to predict effective rice panicles per unit, which is labor-intensive, inefficient, and error-prone (Madec et al., 2019; Zhao et al., 2019). Therefore, an efficient and accurate method for automatic detection and counting of rice panicles is necessary.

The application of rice panicle recognition technology in agricultural production under field conditions is limited by the accuracy of rice panicle recognition in complex environments and the detection speed of the model. Deep learning and image processing technology, which can quickly identify the number of rice panicles per unit area, have been widely used in agriculture in recent years (Fu et al., 2020). Rice panicle recognition is primarily divided into image segmentation and target detection. Xiong et al. (2017) proposed a rice panicle segmentation algorithm (panicle-SEG) that can accurately segment rice panicles in different varieties and complex environments. An unsupervised Bayesian approach was used to segment the unmanned aerial vehicle (UAV) rice panicle images of different varieties and panicle types during the tasseling period with a mean F1 score of 82.10%. However, this method was only applied to upright panicles (Hayat et al., 2020). Zhou et al. (2019) proposed an improved region-based fully convolutional network (R-FCN) algorithm for UAV rice panicle image recognition with an F-measure of 87.4%. However, this method had limitations in training time and image background. Additionally, only one type of rice panicle was used for testing, which limited its application. Yang et al. (2020) used the FPN-Mask (feature pyramid network mask) method to segment rice panicles with an accuracy of 0.99; however, the effect of rice panicle type on segmentation accuracy was not considered. Shao et al. (2021) proposed a localization-based FCN combined with a watershed algorithm for dense rice panicle recognition and counting, with an accuracy of 89.88%. The aforementioned study that uses image segmentation for the actual panicle detection counts, as well as for investigating the effect of spike type on panicle detection, has limitations.

Compared with the traditional image segmentation techniques, the deeper features of rice panicles in the complex field environment can be extracted by using deep learning target detection. You only look once version 4 (YOLOv4) is a representative deep learning model with high speed and accuracy, which is widely used in agriculture, including crop and fruit detection (Yang et al., 2021; Sozzi et al., 2022; Wang et al., 2022a), disease identification (Roy et al., 2022), and lightweight model deployment (Li et al., 2022). However, it is rarely applied to panicle recognition. Compared with two-stage models, such as faster regions with convolutional neural network (Faster-RCNN), the YOLOv4 target detection model offers enhanced detection accuracy while reducing model size, which makes it suitable for future mobile deployment. A feature pyramid-based rice panicle detection method was proposed based on the images Nanjing 46 rice varieties in small-scale complex field environments. This method achieved a recall rate and accuracy rate of 90.82% and a accuracy rate of 99.05%, respectively (Jiang et al., 2020). This indicates the algorithm’s ability to recognize small-sized rice panicles for local occlusion. However, the algorithm was not developed considering UAV and is limited for large-scale applications. The improved Faster-RCNN algorithm was proposed and used to identify rice panicles in potted conditions, and the mAP of this algorithm achieved 80.3% (Zhang et al., 2021). However, this method is designed to detect rice panicles under pot conditions and cannot be directly applied to complex field environments. A multi-scale hybrid window rice panicle detection method was proposed to detect panicles of Nanjing 46 rice varieties at the maturity stage and afforded better robustness for high-density rice panicle counting (Xu et al., 2020). However, when the number of rice panicles in the images increased to 71–80, the recognition accuracy decreased to 86.9%. Zhang et al. (2022a) improved Faster-RCNN to identify multi-growth period rice panicles and the mAP reached 92.47%. However, there were difficulties in actual field testing for mobile applications. Wang et al. (2022b) proposed a new method to remove repeated detections and achieved an accuracy of 92.77%. The methods, however, need to be optimized for UAV images and different density identification.

Although UAV photography considerably improves the efficiency of image acquisition (Oktay et al., 2018; Zhang et al., 2022b), the shape of panicles, occlusion, overlap, background changes, and reduced image quality due to the high density of rice panicles, light intensity, as well as differences in varieties can pose additional difficulties for the model in recognizing rice panicles (Zhao et al., 2021). In particular, the presence of sword leaf shading and scattered rice panicle overlap reduces accuracy of rice panicle detection. Therefore, improving the recognition of different spike types using a model in complex environments remains a challenge. Most of the current research focuses on the improvement of algorithm accuracy and the upright spike type of Japonica rice (Jiang et al., 2020; Xu et al., 2020). Few studies (Zhang et al., 2021) have focused on identifying different varieties and curved panicle types of hybrid Indica rice using UAV images in large-scale complex environments.

To address these challenges, this study establishes a universal model for curved rice panicle identification considering different Indica hybrid rice varieties from multiple regions in the Sichuan province. Images of multiple varieties of rice panicles were acquired using UAV from different ecological points. The model was trained to detect rice panicles based on the improved YOLOv4 model. MobileNetv2 was introduced to replace CSPDarkNet53 as the backbone YOLOv4 feature extraction module to make the model lightweight. Moreover, the convolutional block attention module (CBAM) attention mechanism and focal loss function were introduced for accurate recognition of rice panicle images in a mixed dataset. Finally, soft non-maximum suppression (soft-NMS) was utilized to address the dense shading of similar samples. The enhanced detection performance of this model enables its use as a general detection counting model for different varieties, ecological regions, and types of curved rice panicles in complex environments, making it a useful tool for rice yield prediction and identification of rice panicles.

## 2 Materials and methods

### 2.1 Experimental materials

**Figure 1:**
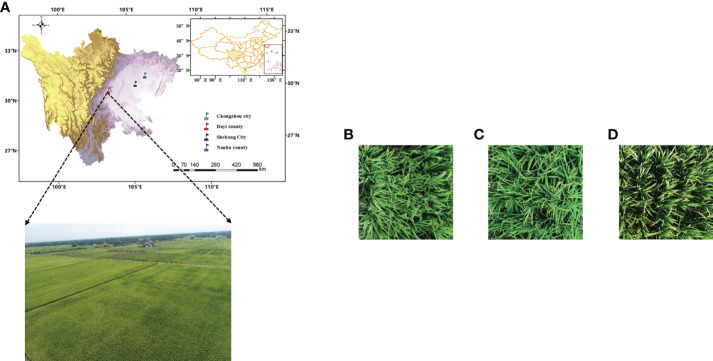
Experimental site and photos of different types of rice panicles. Experimental site of Dayi County **(A-D)** represent spike above leaf, leaf above spikes, and middle type, respectively.

The images of rice panicles were collected in the demonstration areas of high-yield rice production at four different ecological sites in the Sichuan province: Dayi County, Shehong City, Nanbu County, and Chongzhou City (**Figure 1A**). Sichuan is located in a subtropical monsoon climate zone. The location and pattern of the image collection are shown in **Figure 1A**. Different types of rice panicles—spike-above leaf, leaf-above spike, and middle types—were used as testing materials. The rice varieties and classification of types for each testing location are listed in **Table 1**. The UAV images of the three types of materials are shown in **Figures 1B-D**, respectively. Cultivation management measures of different rice varieties in different ecological sites are shown in **Table S1**.

**Table 1:** Experimental rice varieties and their classifications.

Study area	Leaf above spike	Spike above leaf	Middle type
Dayi County	Shen9you28 Chuankangyousimiao Chuanyou6709 Quanyou822	Jingliangyou534	Chuankangyou2115 Yixiangyou2115
Nanbu County		Tianyouhuazhan JingliangYou534	
Chongzhou City			Yixiangyou2115
Shehong City	Chuanzhongyou3877	Jingliangyou534	Chuankangyou2115

### 2.2 UAV image acquisition

A UAV (DJI PHANTOM 4 RTK) was used to collect large-scale images of rice panicles under complex field environments. Under actual field production conditions, rice panicles are easily blocked by leaves. Therefore, to reduce the detection error of rice panicles, images were acquired seven days after the full heading stage in early August 2021. Another set of images were collected at maturity stage in mid-September to ascertain the suitable period for the detection of different types of rice panicles. Clear and cloudless weather conditions were selected for image collection (9:00–11:00 am, 3:00–5:00 pm). The manual flight mode was used with a flight level of 3 m and gimbal tilt angle of 90°. The collected rice panicle images were uniformly 5742 × 3648 pixels in size. In addition, we set up an independent dataset (Jingyou781) in the experiment to verify the model. The independent validation set Jingyou 781 was collected from the experimental field with different panicle fertilizers (**Table S2**) in Dayi County. The collection time and other factors were kept consistent for all images. The remaining camera settings were: ISO-Automatic, Aperture- f/2.2, Focal length-5.74mm.

### 2.3 Data annotation

**Figure 2:**
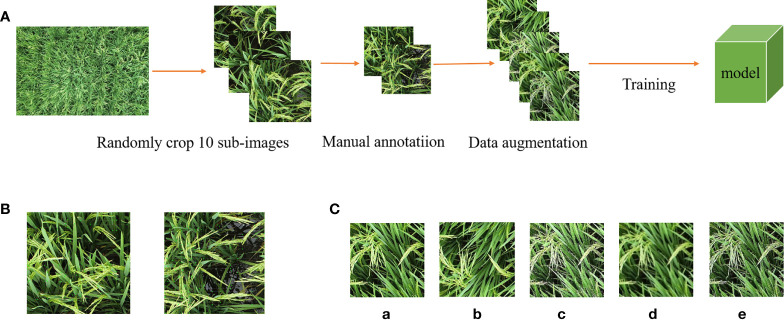
Data pre-processing process. **(A)** Image pre-processing process. **(B)** Example of data labeling. **(C)** Panicle image enhancement (a-e) Original image; flip up and down; image sharpening; Gaussian noise; e: Emboss filter.

The field environment had a significant influence on the detection of the panicles. Data associated with multiple varieties of genotypes and ecological points were included in the panicle image dataset. The accurate identification of rice panicles was hindered by the inconsistent and scattered positioning of the panicles. The leaf-above type panicles were particularly difficult to identify as the panicles are naturally hidden beneath the leaves. The image and processing flow chart are shown in **Figure 2A**. To reduce the cost of data processing annotation and model training time, the original rice panicle images were cropped randomly to 10 images of 608 × 608 pixels using MATLAB (2018b, The MathWorks, USA). Then, for the pre-processed images, the open-source software LabelImg was used for rice panicle labeling (GitHub - tzutalin/labelImg, 2022). For overlapping rice panicles, only the exposed parts were marked. The annotation category label is rice panicle (**Figure 2B**), and the annotation information was saved in the form of a Pascal VOC dataset. Finally, the dataset was amplified by up-and-down, adding noise, emboss filter and sharpening, as shown in **Figure 2C**. There were 10,285 images after image enhancement, including 8,330 images in the training set, 926 images in the validation set, and 1,029 images in the test set.

### 2.4 Rapid detection method for curved rice panicles

#### 2.4.1 YOLOv4 model

Based on the initial YOLO family of networks, YOLOV4 was optimized to varying degrees in terms of model training, activation functions, loss functions and backbone networks. As a two-stage representative network, Faster-RCNN is characterized by low recognition error rate and high accuracy (Ren et al., 2016). Compared to the Faster-RCNN two-stage algorithm, YOLOv4 has a better balance of speed and accuracy, a significant improvement in detection speed, and is widely used in agriculture (Bochkovskiy et al., 2020). The YOLOv4 model primarily consists of the CSPDarknet53 backbone feature extraction network, spatial pyramid pooling (SPPNET), path aggregation network (PANET), and YOLO-Head modules, which generate the coordinates, width, and height, of the candidate frames and final rice panicle prediction frame. CSPDarknet53 comprises several residual modules, which are composed of CSP-X and CBM modules stacked on top of each other. Furthermore, SPPNET can significantly improve the size of the receptive field and extract the most salient contextual features (He et al., 2015). In addition, PANet improves the bottom-up strategy to construct feature pyramids, which can achieve improved feature extraction for targets of different scales and sizes.

#### 2.4.2 Improvement of the YOLOv4 model

**Figure 3:**
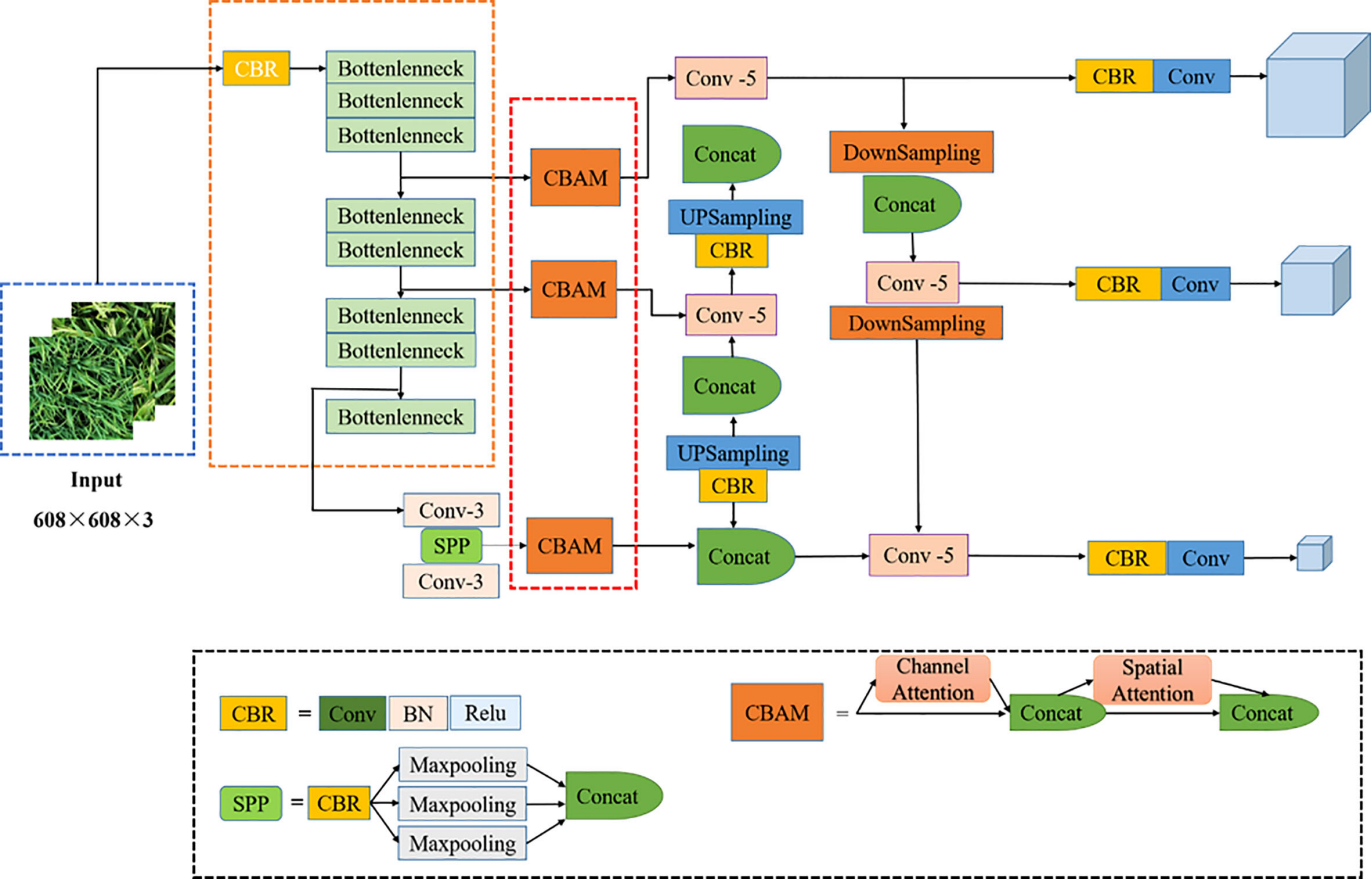
Improved YOLOv4 model structure. The dashed boxes represent improvements to the module. SPP and CBAM represent the SPPNET and attention mechanism modules respectively, Conv represents the convolution operation.

YOLOv4 stacks multiple residual modules in the backbone extraction network CSP-Darknet53, resulting in numerous model parameters. This limits the further application of the rice panicle recognition model in agriculture. We aimed to further improve the detection accuracy and speed of this research method for curved rice panicles, with the complexity of the mixed datasets of UAV images of different ecological zones, varieties, and rice panicle types in the complex field environment. To this end, we propose the lightweight MobileNetV2 as the backbone feature extraction network. The CBAM is added to the image feature fusion stage, and soft-NMS is used to handle dataset occlusion as some of the curved panicles overlap and block each other approximately 7 days after the full heading stage. Further, focal loss was used to optimize the category loss function of the original YOLOv4 model. The model architecture of the improved rice panicle detection network is depicted in **Figure 3**.

#### 2.4.3 Improvement of the YOLOv4 backbone

The YOLOv4 network has better detection accuracy and detection speed (Li et al., 2021). Although the YOLOv4 backbone network CSPDarknet53 can effectively extract depth feature information, the limitation of the number of parameters and computational resources leads to difficulties in applying it in practical agricultural production. Therefore, we improved the original YOLOv4 model to make it more embeddable into mobile devices in the future.

**Figure 4:**
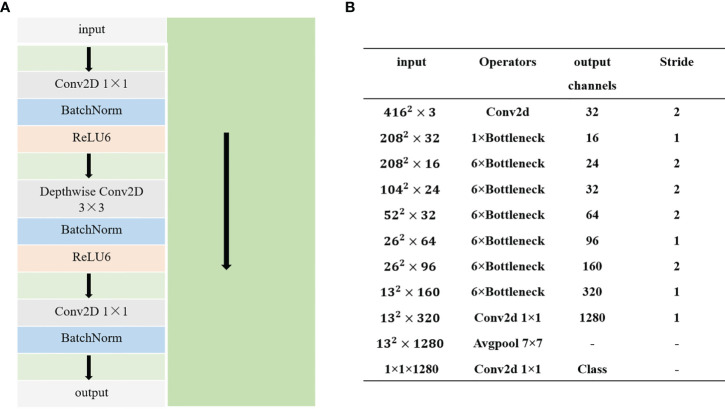
Inverse residual structure **(A)** and structure of MobileNetv2 **(B)**.

In this study, MobileNetv2 was used to replace CSPDarkNet53, which is the backbone feature extraction network of the YOLOv4 model. MobileNetv2, a lightweight feature extraction network, is an improved version of MobileNet, which uses the depthwise convolution module of MobileNetv1 and prevents the destruction of the RELU6 function when applied to the features of a low-dimensional rice panicle. The linear bottleneck structure is introduced instead of RELU6 and then combined with the inverse residual module to form the MobileNetv2 network. The inverse residual structure is proposed by combining the depth-separable convolution, linear bottleneck structure, and residual network to enhance the accuracy of the algorithm. The inverse residual and overall structures of MobileNetv2 are shown in **Figures 4A, B**, respectively. The left part of **Figure 4A** represents the backbone, and the right green part represents the residual structure, which connects the input to the output directly. The inverse residual structure lifts the low dimension of the input by a 1×1 convolution, and a depthwise convolution is used to extract the features. Finally, a 1×1 convolution is used for dimensionality reduction. **Figure 4B** shows the overall MobileNetV2 network structure; Conv2D represents the convolution operation, bottleneck represents the inverse residual module, and Avgpool is the global pooling operation.

#### 2.4.4 Attention module

The attention mechanism is divided into spatial, channel, and mixed spatial and channel attention mechanisms. In this study, CBAM (Woo et al., 2018) is the combined channel and spatial attention mechanism, which was inserted into the feature-enhanced network module in the YOLOv4 model. CBAM is an efficient module with negligible computational overhead and is given an intermediate feature mapping layer as input. The CBAM attention mechanism was used to assign more weight to the rice panicle’s feature region in the image through the spatial and channel learning of rice panicle features and focuses on the extraction of important features of rice panicles during training. It also suppressed distracting factors such as rice leaves and water reflection in the field to improve the accuracy of the model. The channel and spatial attention mechanism expressions of CBAM are given in the following equations:


(1) $$Mc\left(F\right)=\sigma \left(\text{MLP}\left(\text{AvgPool}\left(F\right)\right)+\text{MLP}\left(\text{MaxPool}\left(F\right)\right)\right)=\sigma \left(W_{1}\left(W_{0}\left(F_{\text{avg }}^{c}\right)\right)+W_{1}\left(W_{0}\left(F_{\text{max }}^{c}\right)\right)\right);$$

(2) $$Ms\left(F\right)=\sigma \left(f^{7\times 7}\left(\left[F_{\text{avg }}^{s};F_{max}^{s}\right]\right)\right);$$

where MLP is a multilayer perceptron with a hidden layer, σ is the sigmoid operation and the convolution kernel size is 7 × 7. $\;F_{\text{avg }}^{c}\text{ and}\;F_{\text{max }}^{c}$ represent average-pooled features and max-pooled features. *W*_0_ ∈ ℝ^*C*/*r*×*C*^, *W*_1_ ∈ ℝ^*C*/*r*×*C*/*r*^ indicates the weight of MLP.

#### 2.4.5 Soft-NMS

This study focused on different types of bent rice panicles, which exhibit different degrees of shading. Owing to the density of the rice plants in the images, the overlapping of leaves or panicles causes an obstruction which reduces the detection accuracy of the model. NMS typically misses certain rice panicles because of overlapping and is not suitable for rice panicle detection using UAV images. By contrast, Soft-NMS can significantly improve the recognition rate of the model in the presence of occlusion (Bodla et al., 2017). Therefore, Soft-NMS was introduced in the YOLOv4 model instead of NMS. Soft-NMS accounts for both the score and degree of overlap as follows:


(3) $$s_{i}=\{\begin{array}{c} s_{i},\;\;\;\;\;\;\;\;\;\;\;\;\;\;\;\;\;\;\;\;\;\;\;\;\;\;\;Iou\left(M,b_{i}\right)<N_{t}\\ s_{i}\left(1-\text{Iou}\left(M,b_{i}\right)\right),\;\;\;\;\;\;\;\;\;\;Iou\left(M,b_{i}\right)\geq N_{t} \end{array}$$

where *s_i_* is the final score, i is the subscript, M is the box with the highest score in the prediction box set, *b_i_* is the box in the prediction box set B, and *N_t_* is the intersection-over-union (IoU) threshold of M and *b_i_*.

#### 2.4.6 Improvement of the loss function

The loss functions, including the complete intersection over union (CIOU), classification, and confidence losses, were used in the YOLOv4 model. CIOU loss also considers the overlap area, centroid distance, and aspect ratio of the bounding box regression. The original loss function was the crossover loss function, which was calculated as follows:


(4) $${ℒ}_{CIoU}=1-IoU+\frac{\rho^{2}\left(b,b^{gt}\right)}{c^{2}}+\alpha \mathrm{v;}$$

(5) $${ℛ}_{\text{CIoU}}=\frac{{\text{ρ}}^{2}\left(b,b^{gt}\right)}{{\text{c}}^{2}}+\text{αv};$$

(6) $$\text{v}=\frac{4}{{\text{π}}^{2}}{\left(\text{arctan}\frac{{\text{w}}^{\text{gt}}}{{\text{h}}^{\text{gt}}}-\text{arctan}\frac{\text{w}}{\text{h}}\right)}^{2};$$

(7) $$\text{α}=\frac{\text{v}}{\left(1-\text{IoU}\right)+\text{v}}\;;$$

where *ρ* ^2^(*b*,*b* ^*gt*^)  represents the Euclidean distance between the center points of the predicted and real boxes, respectively; c represents the diagonal distance that can contain both the prediction box and true box minimum closure region; v represents the aspect ratio parameters; and α represents the positive trade off parameters. Further, w and h denote the width and height of the prediction box, respectively, and *w^gt^* and *h^gt^* denote the width and height of the real box, respectively.

In the curved rice panicle data set, the model ignored samples that are difficult to classify when there are overlaps and rice panicle occlusions in the image. Hence, the appropriate loss function must be selected to balance the contribution of positive and negative samples to the total loss. Therefore, by improving the loss function, the model network can focus more on the samples that are difficult to classify. The focal loss equation is expressed as follows:


(8) $${\text{L}}_{\text{fl}}=\{\begin{array}{c} -{\left(1-\hat{\text{p}}\right)}^{\text{γ}}log\left(\hat{\text{p}}\right)\text{ if }y=1\;\\ -{\hat{\text{p}}}^{\text{γ}}log\left(1-\hat{\text{p}}\right)\text{ otherwise} \end{array}$$

Here, $\hat{p}$ represents the probability of correctly classifying a rice panicle. The classification loss function of the original YOLOv4 model is optimized using the focal loss without increasing the computational overhead of the model. Note that $1-\hat{p}$ approaches 1 as $\hat{p}$ decreases, indicating that the overall loss has a negligible effect on accuracy. Thus, replacing the category loss function with the focal loss function enables the model to focus on rice panicles with overlapping occlusion in the image.

#### 2.4.7 Model training

**Figure 5:**
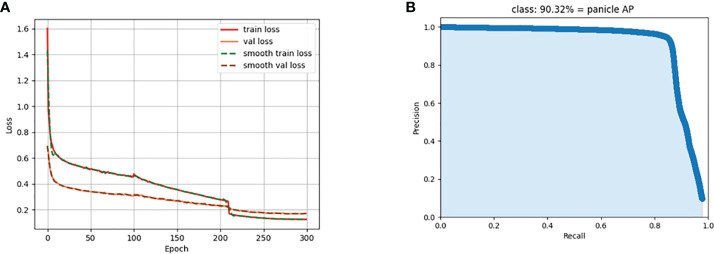
**(A)** Training loss curve and **(B)** P-R curve of panicle detection for the proposed method.

Processing was performed using an AMD 5900X CPU, 32GB memory, Windows 10, RTX3060 GPU, 12G video memory, the operating environment was PyTorch 1.7.1, Python 3.7, CUDA 11.0. The model training is based on the transfer learning technique to speed up the model convergence. The k-means clustering algorithm was used to generate the anchor coordinates of (25, 21), (32, 43), (54, 28), (63, 49), (42, 73), (101, 51), (70, 97), (135, 81), and (111, 136). Mosaic data augmentation, label smoothing, cosine smoothing, cosine annealing decay, and other training techniques were used in the training process to improve model accuracy. The other training parameters are shown in **Table 2**. The training loss of the improved model is shown in **Figure 5A**. The model training loss decreases and converges as the number of iterations increases. The model converges by the 300^th^ epoch, and the model loss value is 0.124. The influence of label smoothing training techniques on model loss is compared in **Figure 5A**. Using label smoothing techniques improves the generalization ability of the model. **Figure 5B** shows the P-R plots of the improved model; the area enclosed under the curve represents the AP value of the model.

**Table 2:** Network training hyperparameters.

Parameters	Set value
Optimizer	SGD
Momentum	0.9
Initial learning rate	1×10^-2^
Label smoothing	0.01
Epoch	300
Decay weights	5×10^-4^

#### 2.4.8 Evaluation metrics

In this study, precision (P), recall (R), mean average precision (mAP), F1-score, detection speed, and detection time of the model were calculated to objectively evaluate the detection effect of this model for curved rice panicles in a complex field environment. In the experiment, IOU greater than 0.5 was defined as a positive sample. By definition, P, R, mAP, and the F1-score can be expressed as


(9) $$P\left(\%\right)=\frac{T_{P}}{T_{P}+F_{P}}\times 100;$$

(10) $$R\left(\%\right)=\frac{T_{P}}{T_{P}+F_{N}}\times 100;$$

(11) $$\text{F}1=2\times \frac{PR}{P+R};$$

(12) $$\;mAP=\int_{0}^{1}P\left(R\right)dR;$$

where TP, FP, TN, and FN represent true positive, false positive, true negative, and false negative, respectively, based on the true and predicted classes of the target object. mAP is the average of AP across the total number of classes, which indicates the detection performance of the model for rice panicles; higher mAP values represent better models. The model detection speed is evaluated by frames per second (FPS) transmission, and the larger the attained FPS, the better the model fluency and the faster the detection speed.

In addition, quantitative model count accuracy metrics including root-mean-square error (RMSE) and R^2^ were used. The lower the RMSE, and the larger the R^2^ value, the better is the model performance. They are expressed as follows:


(13) $$R^{2}=1-\frac{\sum_{i=1}^{n}{\left(t_{i}-c_{i}\right)}^{2}}{\sum_{i=1}^{n}{\left(t_{i}-{\bar{t}}_{i}\right)}^{2}}$$

(14) $$\text{RMSE}=\sqrt{\frac{1}{n}\sum_{i=1}^{n}{\left(t_{i}-c_{i}\right)}^{2}}$$

(15) $$\text{Statistical accuracy}=1-\left|\frac{Predict-True}{True}\times 100\%\right|.$$

## 3 Results

### 3.1 Ablation study

The results of the ablation experiments showed the effectiveness of the improved model, as shown in **Table 3**. The model achieved a mAP of 82.92%, only by replacing the MobileNetv2 backbone feature extraction network. Using the CBAM attention mechanism module to join in the feature fusion stage, the model mAP reached 83.10%. Then, using Soft-NMS to replace NMS resulted in a mAP of 87.02%. Finally, focal loss is used to replace the category loss function in the YOLOv4 loss function to improve the detection effect of the model for identifying samples with overlapping rice panicles, increasing the mAP to 90.32%.

**Table 3:** Ablation study.

MobileNetv2	CBAM	Soft-NMS	Focal loss	mAP (%)
**✓**				82.92
**✓**	**✓**			83.10
**✓**	**✓**	**✓**		87.02
**✓**	**✓**	**✓**	**✓**	**90.32**

The symbol "✓" means adding an improvement strategy.

### 3.2 Detection effect of improved models

Compared to the MobileNetv2-YOLOv4 model, the mAP of MobileNetv2-YOLOv4-DepthwiseConv decreased by 4.79% when the model training parameters were kept consistent, as can be observed in **Table 4**. Compared with Our method and the YOLOv4 model, the mAP of Our-method-Depthwise-Conv, a feature enhancement module using depthwise convolution, decreased by 9.56% and 3.36%, respectively. Furthermore, the detection time of Our-method-Depthwise-Conv only increased by 0.016s compared to Our Method. These results showed that although the detection speed of Our Method was slightly reduced, the detection accuracy was significantly improved.

**Table 4:** Experimental results of rice panicle recognition with different target detection models.

Model	mAP (%)	Test time (s)	FPS
MobileNetv2-YOLO	82.92	0.161	62.13
MobileNetv2-YOLO4-Depthwise Conv	78.73	0.0152	65.95
Our-method-Depthwise Conv	80.76	0.0209	47.92
Our method	90.32	0.0225	44.46

The rice panicle dataset is a hybrid dataset of UAV images, composed of different varieties, panicle types, and ecological points in a complex environment in a large field. The rice panicle detection model mAP in this study reached 90.32% of our method compared with the YOLOv4 model and Faster-RCNN (**Table 5**). Our improved model in this paper has the highest mAP, F1, and recall, which increased by 6.2%, 0.12, 16.24% respectively, as compared to the original YOLOv4 model. With respect to the Faster-RCNN model, our improved model increased the same values by 45.68%, 0.50, 29.24%, respectively. The test time of our model was the lowest, which decreased by 0.0036–0.0655 s, compared with the other models. The above results indicate that our improved model performs better than previous models in terms of detection accuracy with slightly decreased but comparable speed for the identification of curved rice panicles in a complex field environment.

**Table 5:** Recognition effects of different advanced target detection models.

Model	mAP (%)	F1	Recall (%)	Test time (s)	FPS
YOLOv4	84.12	0.77	66.12	0.0261	38.32
Faster-RCNN	44.64	0.49	53.12	0.0880	11.31
Our method	90.32	0.89	82.36	0.0225	44.46

### 3.3 Recognition effect of different types of rice panicles

**Figure 6:**
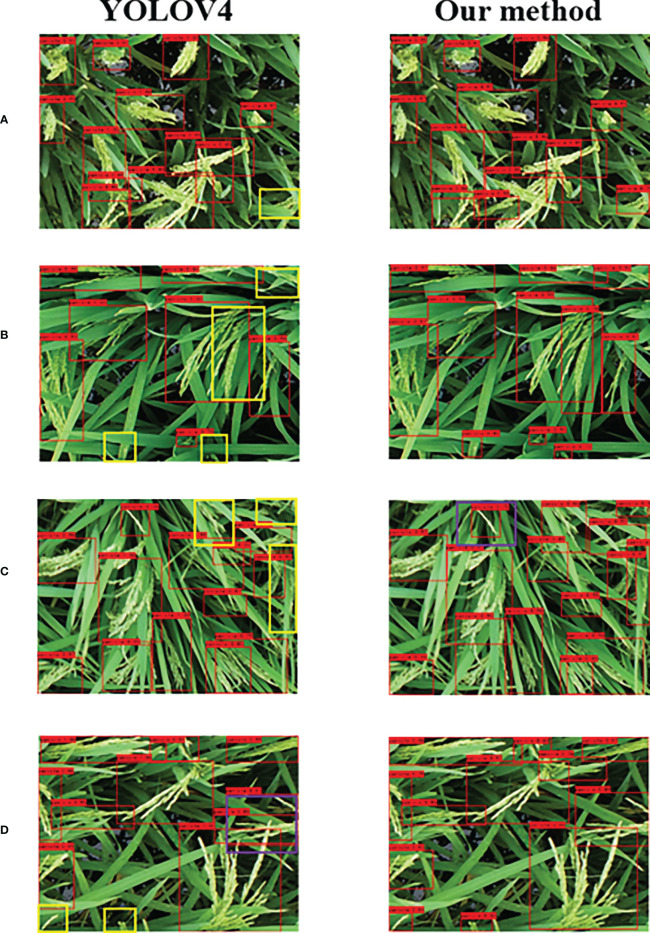
Recognition effects of YOLOv4 model (left) and the improved algorithm in this study (right). **(A)** Independent dataset recognition; **(B)** leaf-above spike type; **(C)** spike-above leaf type; **(D)** middle type. The yellow and purple boxes represent missed and false detection, respectively.

As shown in **Figure 6**, the independent dataset of Jingyou781 varieties was used for model validation (**Figure 6A**). The YOLOv4 model showed misrecognition in the presence of occlusion, and our method performs better in the independent dataset. The leaf-above spike type, scattered spike type, and small target panicles were all missed in the original YOLOv4 algorithm, while our method did not misidentify the small scattered panicle type in the image (**Figure 6B**). The spike-above leaf type showed more scattered panicle types, leading to false recognition in both the YOLOv4 model and our method (**Figure 6C**). For the middle type rice panicle (**Figure 6D**), YOLOv4 misrecognized the small target panicle owing to the different extraction time in the actual field environment; however, the recognition of our improved algorithm showed excellent performance.

### 3.4 Accuracy validation of model counting

The improved model yielded better detection performance for the three types of curved rice panicles (**Table 6**). One hundred images of each type from the training set were randomly selected, and the model was used to identify and compare the images with that of the actual labeled frames. Results showed that for the middle, spike-above leaf, and leaf-above spike types of panicles, the R^2^ value was 0.84, 0.89, and 0.92 respectively, and the RMSE was 2.56, 1.95, and 4.39, respectively. The larger RMSE of the leaf-above spike type is because of the presence of more panicles obscured by leaves in the image, resulting in more rice panicles being missed. The manual labeling of the three types of rice panicles in the 100 images found 1589, 1961, and 3510 panicles, respectively. Comparatively, our improved model recognized 1699, 1965, and 3754 panicles, respectively. The original YOLOv4 model recognized 1325, 1518, and 2505. Thus, the accuracy of our method was 93.08%, 99.80%, and 93.05% for the three types of rice spike counts, respectively. The accuracy of YOLOv4 for the three types of rice spikes was 83.39%, 74.41%, and 71.37%, respectively. The original YOLOv4 model performed the worst in the leaf-above spike type count.

**Table 6:** The accuracy of our method for identifying different types of panicles.

Type	Seven days after the full heading stage			Mature stage		
	*R* ^2^	RMSE	rRMSE	*R* ^2^	RMSE	rRMSE
Middle type	0.84	2.56	0.1741	0.46	2.77	0.4567
Spike above leaf	0.89	1.95	0.0986	0.38	2.86	0.4321
Leaf above spike	0.92	4.39	0.1520	0.31	3.28	0.4097

### 3.5 Model accuracy in different periods

**Figure 7:**
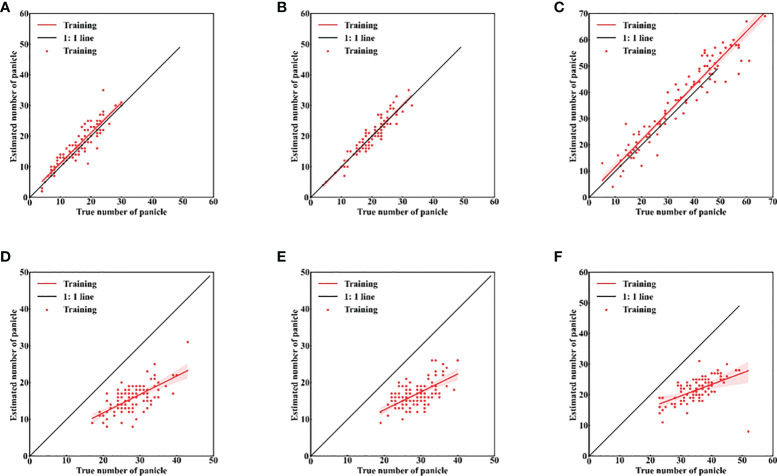
Accuracy of rice panicles identification in different data sets. **(A–C)** seven days after the full heading stage; **(D–F)** mature stage; **(A, D)** middle type; **(B, E)** spikes above leaves type; **(C, F)** leaves above spikes type.

The maturity stage of the rice crop when the images were taken also had a significant impact on the accuracy of the models. This is due to the presence of more bent and dispersed rice panicles, and the fact that both leaves and panicles turn yellow at maturity. This also had an impact on manual labeling. In this study, 514 images of different varieties of rice at different ecological points were labeled at maturity. After data enhancement for model training, the mAP of the model had only 79.66% at maturity stage (**Table 7**). This result indicated that data collection is more appropriate at 7 days after the full heading stage for the identification and counting of rice panicles. A comparison of 100 randomly selected marker images of the three rice panicle types with the number predicted by the model is shown in **Figure 7**. The model performed poorly in identifying the three types at maturity, with R^2^ values of only 0.46, 0.38, and 0.31, for the middle, spike-above leaf, and leaf-above spike types of panicles, respectively.

**Table 7:** Influence of sampling period on model accuracy.

Stage	mAP (%)	F1	Recall (%)	Number of training images	Number of images after data enhancement
Seven days after the full heading stage	90.32	0.89	82.36	2056	10280
Mature stage	79.66	0.75	62.05	514	2570

## 4 Discussion

Most rice panicle identification studies focus on upright spikes and potted plants. Our study targeted the curved spike type of Indica rice in the actual field production environment, which significantly increased the difficulty of recognition. Compared to the method of Zhang et al. (2021), our method’s mAP was 10.02% greater, and the detection speed improved by 146.6 ms (**Table S4**). In a previous study, the addition of the CBAM attention mechanism for YOLOv4 improved wheat detection accuracy in the presence of occlusion (Yang et al., 2021). Compared with the recognition of rice panicles using UAV images based on improved R-FCN (Zhou et al., 2019), our method improves the recognition accuracy of different varieties by 3.52%. The mAP of our method improves by 45.68% and 6.2% compared to the Faster-RCNN and YOLOv4 models, respectively (Ren et al., 2017). The above results indicate the better performance of our improved model in the mixed dataset. The weight ratio of fertilizer formulations are shown in **Table S2**. In addition, the results of the panicle count for different fertilization treatments for the independent dataset demonstrates the feasibility of our method to build a universal model for the mixed dataset (**Table S3**). The mAP of the proposed method achieves 90.32% while accounting for the speed of detection (**Table 4**). This experiment shows that the proposed method effectively addresses challenges associated with different varieties, panicle types, and ecological regions to achieve accurate rice panicle identification and counting.

The type of the rice panicle and the variety of the rice crop influenced the accuracy of model detection (Xu et al., 2020). We established a general method by collecting different varieties at the same period, and the mAP value of the model was 90.32% (**Table 4**). The results from the independent dataset counts in **Table S3** showed that it is feasible to develop a generic rice panicle detection model for different varieties in the same period. However, in the G-BLACK processing, because the heads of the panicles are scattered, there were many repeated detections.

**Figure 8:**
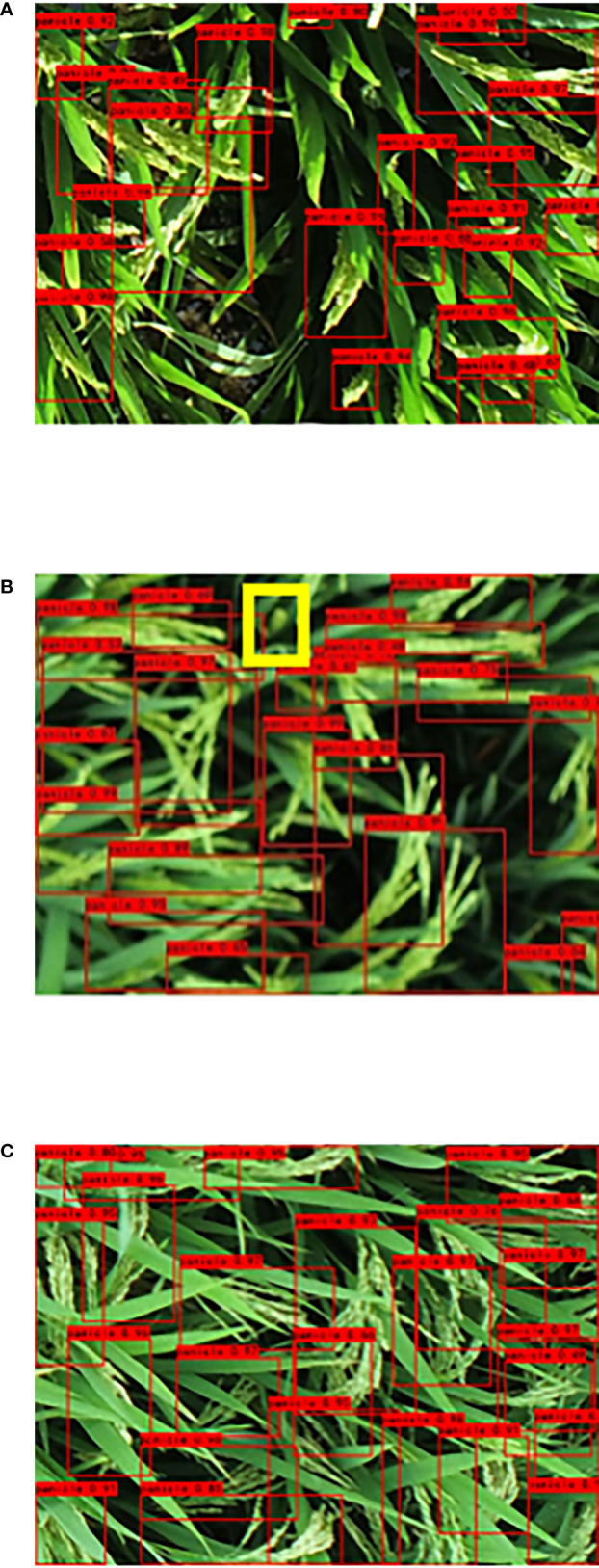
Effect of different complex environments on the recognition of scattered panicle in a large field. **(A)** Image taken using strong exposure; **(B)** UAV image of canopy disturbance; **(C)** Scattered panicle in complex environment.

The photo environment also affects the image quality, and thus affects the recognition accuracy of the rice panicles. **Figure 8** shows the effect of this study’s improved algorithm on rice panicle recognition under different complex environmental disturbances. For example, there are inconsistent light conditions when using a UAV for rice panicle image acquisition. When the improved algorithm was used for the recognition of rice panicles under strong light conditions, there were no misrecognitions (**Figure 8A**). Due to the canopy disturbance caused by the UAV rotor, the image is blurred and model detection is affected, leading to the lack of recognition of spikelets under the leaf shade in the proposed method (**Figure 8B**). The proposed method is based on the existence of dispersed rice panicle types in the image, which can easily cause multiple recognition for dispersed spike types (**Figure 8C**). Building a universal detection model for dispersed-type rice panicles in a complex field environment is complex, as the scattered state of the rice panicles at 7 days after full heading has an impact on the integrity of the other rice panicles, resulting in misidentification and misrecognition.

**Figure 9:**
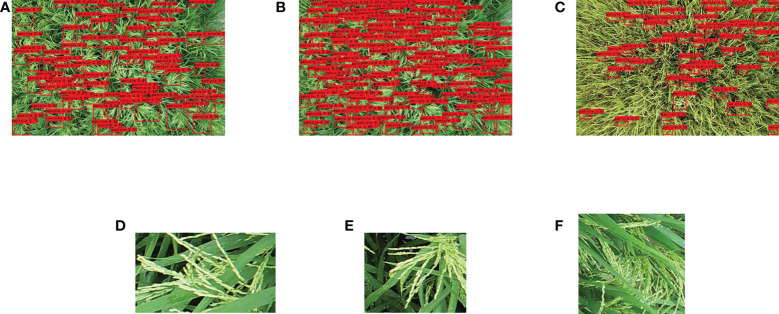
Recognition results of UAV images using the large-scale dispersion spike model. **(A)** Recognition results of the original YOLOv4 model, and **(B)** Recognition results of the improved algorithm **(C)** Improved algorithm recognition during maturity. **(D–F)** Large-scale UAV view prediction frame analysis.

To further explore the possibility of generalizing the improved model for image detection in a wide field of UAV view (**Figure 9**), this study discussed the rice panicle images from a UAV at a distance 3 m from the rice canopy. The UAV view improvement algorithm did not achieve better results at maturity because of the increase in dispersed spike types at this stage (**Figure 9C**). The presence of scattered rice panicles in the image leads to multiple model recognitions, which makes it difficult to maintain the integrity of the rice panicles (**Figure 9D**). Then, the improved model was employed to detect and evaluate the collected image. The poor recognition of dispersed rice panicles photographed by a UAV is mainly caused by the following reasons: the obscuration of sword leaves (**Figure 9D**), small rice panicles in the UAV image (**Figure 9E**), and multiple dispersed rice panicles interacting with each other (**Figure 9F**). For scattered rice panicles, the more accurate and easier identification period should be selected by counting the dynamic changes of rice panicles. Future research will focus on establishing a specific recognition model that can be combined with the collection of images during a specific period of time in order to establish accurate detection of rice panicles. To achieve large-scale application, lightweight and universal models must be developed. In addition, we must establish a single model to detect the dispersed panicle type in the curved panicle type to reduce misidentification by the model.

## 5 Conclusion

In this study, a universal rice panicle detection model was developed using a mixed dataset to identify panicle images of different Indica hybrid rice varieties grown in different ecological regions in large-scale complex field environments. The improved method outperformed the original YOLOv4 and Faster-RCNN models in terms of detection performance and accuracy for the leaf-above-spike type, spike-above-leaf type, and middle type. The F1 scores improved by 0.12 and 0.40 from those of the two original models, respectively. The detection accuracy of different models at 7 days after full heading stage was significantly higher than that at maturity stage, and the RMSE of the spike-above-leaf type at 7 days after full heading stage was also improved. Different rice varieties were divided into different types for detection and analysis, and all three types obtained improved identification results with our model. The current study illustrates the feasibility of establishing a general rice panicle identification model for a certain period with a mixed dataset of Indica hybrid rice. However, when there are more dispersed panicles in the hybrid Indica rice, a separate rice panicle detection model is needed to improve detection accuracy. In future, we will focus on the detection of scattered spike count and model deployment on mobile devices. The best prediction identification date should be chosen based on dynamic analysis of the three types to build identification models for the characteristics of Indica hybrid rice with more scattered spike types in the future.

## Data availability statement

The original contributions presented in the study are included in the article/**Supplementary Material**. Further inquiries can be directed to the corresponding authors.

## Author contributions

BS: Conceptualization, Writing- Original draft preparation, Data curation, Formal analysis. WZ: Conceptualization, Supervision, Writing- Original draft preparation, Visualization, Software, Writing-Reviewing and Editing. SZ, SH, XY, and ZW: Methodology, Investigation. XL, DY, HX, YC, YT, FD, and HC: Writing- Reviewing and Editing, Resources. WR and XJ: Conceptualization, Supervision, writing-Reviewing and Editing. All authors contributed to the article and approve the submitted version.

## Funding

This work was supported by the Natural Science Foundation of China (grant numbers U20A2022), the Sichuan Science and Technology Program (grant number 2021YJ0492), the Agricultural Science and Technology Innovation Program of the Chinese Academy of Agricultural Sciences, Hainan Yazhou Bay Seed Lab (B21HJ0221), Nanfan special project, CAAS (YJTCY01, YBXM01), and Research and application of key technologies of smart brain for farm decision-making platform (2021ZXJ05A03).

## Acknowledgments

The authors would like to gratefully thank all the members of the Paddy Laboratory of Sichuan Agriculture University of China for their suggestions and help. We would like to thank Editage [www.editage.cn] for English language editing.

## Conflict of interest

The authors declare that the research was conducted in the absence of any commercial or financial relationships that could be construed as a potential conflict of interest.

## Publisher’s note

All claims expressed in this article are solely those of the authors and do not necessarily represent those of their affiliated organizations, or those of the publisher, the editors and the reviewers. Any product that may be evaluated in this article, or claim that may be made by its manufacturer, is not guaranteed or endorsed by the publisher.

## References

[B1] Bochkovskiy A. Wang C. Y. Liao H. (2020). YOLOv4: Optimal speed and accuracy of object detection. arXiv 2004, 10934.

[B2] Bodla N. Singh B. Chellappa R. Davis L. S. (2017). Soft-NMS – improving object detection with one line of code. arXiv 1704, 04503. doi: 10.1109/ICCV.2017.593

[B3] Fu L. Song Z. Zhang X. Li R. Wand D. Cui Y. . (2020). Applications and research progress of deep learning in agriculture. J. China Agric. Univ. 25, 105–120. doi: 10.11841/j.issn.1007-4333.2020.02.12

[B4] GitHub - tzutalin/labelImg. (2022). In: LabelImg is a graphical image annotation tool and label object bounding boxes in images. Available at: https://github.com/tzutalin/labelImg (Accessed 2022/4/29).

[B5] Hayat M. A. Wu J. Cao Y. (2020). Unsupervised Bayesian learning for rice panicle segmentation with UAV images. Plant Methods 16, 18. doi: 10.1186/s13007-020-00567-8 32123536 PMC7035759

[B6] He K. Zhang X. Ren S. Sun J. (2015). Spatial pyramid pooling in deep convolutional networks for visual recognition. IEEE Trans. Pattern Anal. Mach. Intell. 37, 1904–1916. doi: 10.1109/TPAMI.2015.2389824 26353135

[B7] Jiang HY. Xu C. Yao C. Yongkang C. (2020). Detecting and counting method for small-sized and occluded rice panicles based on in-field images. Transactions of the Chinese Society for Agricultural Machinery 51, 152–162.

[B8] Li G. Suo R. Zhao G. Gao C. Fu L. Shi F. . (2022). Real-time detection of kiwifruit flower and bud simultaneously in orchard using YOLOv4 for robotic pollination - ScienceDirect. Comput. Electron. Agric. 193, 106641. doi: 10.1016/j.compag.2021.106641

[B9] Li W. Feng X. Zha K. Li S. Zhu H. (2021). Summary of Target Detection Algorithms. Journal of Physics: Conference Series. 1757, 012003. doi: 10.1088/1742-6596/1757/1/012003.

[B10] Madec S. Jin X. Lu H. De Solan B. Liu S. Duyme F. . (2019). Ear density estimation from high resolution RGB imagery using deep learning technique. Agric. For. Meteorol. 264, 225–234. doi: 10.1016/j.agrformet.2018.10.013

[B11] Oktay T Celik H Turkmen I. (2018). Maximizing autonomous performance of fixed-wing unmanned aerial vehicle to reduce motion blur in taken images. Proceedings of the Institution of Mechanical Engineers, Part I: Journal of Systems and Control Engineering. 232 (7), 857–868. doi: 10.1177/0959651818765027

[B12] Ren S. He K. Girshick R. Sun J. (2016). Towards real-time object detection with region proposal networks. IEEE 6 (2017), 1137–1149. 10.1109/TPAMI.2016.2577031 27295650

[B13] Ren S. He K. Girshick R. Sun J. (2017). Faster r-CNN: Towards real-time object detection with region proposal networks. IEEE Trans. On Pattern Anal. Mach. Intell. 39, 1137–1149. doi: 10.1109/TPAMI.2016.2577031 27295650

[B14] Roy A. M. Bose R. Bhaduri J. (2022). A fast accurate fine-grain object detection model based on YOLOv4 deep neural network. Neural Computing Appl. 34, 3895–3921. doi: 10.1007/s00521-021-06651-x

[B15] Shao H. Tang R. Lei Y. Mu J. Guan Y. Xiang Y . (2021). Rice ear counting based on image segmentation and establishment of a dataset. Plants (Basel) 10, 1625. doi: 10.3390/plants10081625 34451670 PMC8402056

[B16] Slafer G. A. Savin S. Vo. C. (2014). Coarse and fine regulation of wheat yield components in response to genotype and environment. Field Crops Res. 157, 71–83. doi: 10.1016/j.fcr.2013.12.004

[B17] Sozzi M. Cantalamessa S. Cogato A. Kayad A. Marinello F. (2022). Automatic bunch detection in white grape varieties using YOLOv3, YOLOv4, and YOLOv5 deep learning algorithms. Agronomy 12, 319. doi: 10.3390/agronomy12020319

[B18] Wang L. Zhao Y. Liu S. Li Y. Chen S. Lan Y. . (2022a). Precision detection of dense plums in orchards using the improved YOLOv4 model. Front. Plant Sci. 13. doi: 10.3389/fpls.2022.839269 PMC8963500 35360334

[B19] Wang X. Yang W. Lv Q. Huang C. Liang X. Chen G. . (2022b). Field rice panicle detection and counting based on deep learning. Front. Plant Sci. 13. doi: 10.3389/fpls.2022.966495 PMC9416702 36035660

[B20] Woo S. Park J. Lee J. Y. Kweon I. S. (2018) CBAM: Convolutional block attention module. Springer Cham. doi: 10.1007/978-3-030-01234-2_1

[B21] Xiong X. Duan L. Liu L. Tu H. Yang P. Wu D. . (2017). Panicle-SEG: A robust image segmentation method for rice panicles in the field based on deep learning and superpixel optimization. Plant Methods 13, 104. doi: 10.1186/s13007-017-0254-7 29209408 PMC5704426

[B22] Xu C. Jiang H. Yuen P. Zaki Ahmad K. Chen Y. (2020). MHW-PD: A robust rice panicles counting algorithm based on deep learning and multi-scale hybrid window. Comput. Electron. Agric. 173, 105375. doi: 10.1016/j.compag.2020.105375

[B23] Yang B. Gao Z. Gao Y. Zhu Y. (2021). Rapid detection and counting of wheat ears in the field using YOLOv4 with attention module. Agron. (Basel) 11, 1202. doi: 10.3390/agronomy11061202

[B24] Yang Z. Gao S. Xiao F. Li G. Ding Y. Guo Q. . (2020). Leaf to panicle ratio (LPR): A new physiological trait indicative of source and sink relation in japonica rice based on deep learning. Plant Methods 16, 117. doi: 10.1186/s13007-020-00660-y 32863854 PMC7449046

[B25] Zhang Y. Xiao D. Chen H. Liu Y. (2021). Rice panicle detection method based on improved faster r-CNN. Trans. Chin. Soc. Agric. Machinery 52, 231–240. doi: 10.6041/j.issn.1000-1298.2021.08.023

[B26] Zhang Y. Xiao D. Liu Y. Wu H. (2022a). An algorithm for automatic identification of multiple developmental stages of rice spikes based on improved faster r-CNN. Crop J. 10 (5), 1323–1333doi: 10.1016/j.cj.2022.06.004

[B27] Zhang J. Min A. Steffenson B. J. Su W. Hirsch C. D. Anderson J. . (2022b). Wheat-net: An automatic dense wheat spike segmentation method based on an optimized hybrid task cascade model. Front. Plant Sci. 13. doi: 10.3389/fpls.2022.834938 PMC8866238 35222491

[B28] Zhao J. Q. Zhang X. H. Yan J. W. Qiu X. L. Yao X. Tian Y. C. . (2021). A wheat spike detection method in UAV images based on improved YOLOv5. Remote Sens. 13, 3095. doi: 10.3390/rs13163095

[B29] Zhao S. Zheng H. Chi M. Chai X. Liu Y. (2019). Rapid yield prediction in paddy fields based on 2D image modelling of rice panicles. Comput. Electron. Agric. 162, 759–766. doi: 10.1016/j.compag.2019.05.020

[B30] Zhou W. Lv T. Zhang P. P. Huang Y. Ren W. (2016). Regular nitrogen application increases nitrogen utilization efficiency and grain yield in indica hybrid rice. Agron. J. 108, 1951–1961. doi: 10.2134/agronj2016.03.0137

[B31] Zhou C. Ye H. Hu J. Shi X. Hua S. Liu Y. . (2019). Automated counting of rice panicle by applying deep learning model to images from unmanned aerial vehicle platform. Sensors 19, 3106. doi: 10.3390/s19143106 PMC6679257 31337086

