# Supplementary material for: Universal detection of curved rice panicles in complex environments using aerial images and improved YOLOv4 model
Source: Front Plant Sci. 2022 Nov 7;13:1021398. doi: 10.3389/fpls.2022.1021398 (PMC9676644; doi:10.3389/fpls.2022.1021398)
Supplement: Supplementary file 1 [file DataSheet_1.pdf]

Supplementary materials

Table S1| Cultivation measures in the Experimental sites

Experimental sites	Planting measures	Planting specifications	Heading date
**Dayi country**	Machine rice planting	30cm×18cm<br>3-4 seedling/hill	2021.7.31-2021.8.13
**Shehong city**	Machine rice planting	30cm×20cm<br>3-4 seedling/hill	2021.8.08-2021.8.13
**Nanbu country**	Machine rice planting	30cm×16cm<br>3-4 seedling/hill	2021.8.01-2021.8.08
**Chongzhou city**	Machine rice planting	30cm×20cm<br>3-4 seedling/hill	2021.8.07-2021.8.11

Table S2| Weight ratio of fertilizer formulations

Treatment	Urea(%)	Potassium chloride (%)	HA(%)	PASP(%)	GABA(%)
**P-White**	42.6	40.8	/	0.3	/
**G-White**	42.6	40.8	/	/	0.3
**P-Black**	42.6	40.8	4	0.3	/
**G-Black**	42.6	40.8	4	/	0.3

Table S3| Identification results of the independent data set of Jingyou 781 with different fertilizer treatments

	P-White	G-White	P-Black	G-Black	Common spike fertilizer	CK
**Manual count**	324	330	379	395	389	411
**Model count**	378	374	408	499	422	348
**Statistical accuracy (%)**	83.33	86.67	92.35	73.67	**91.52**	84.67

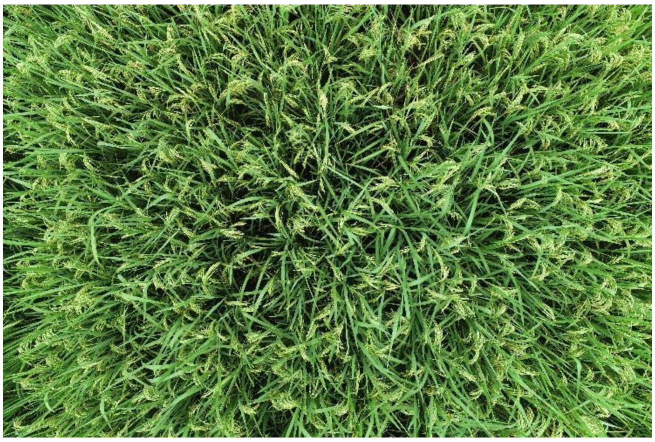

Figure S1|UAV acquisition of raw images

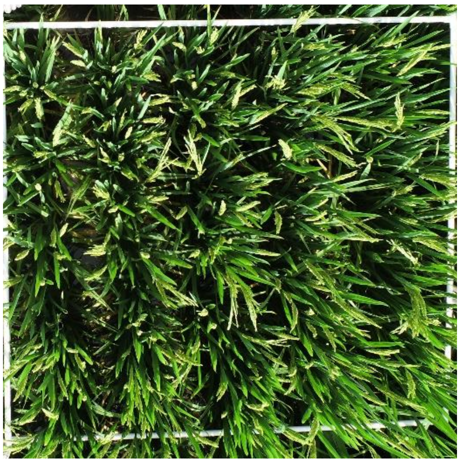

Figure S2 | Example of a boxed area

Table S4 | The result from previous studies on rice panicle detection

Author	mAP (%)	F1-Score	Recall (%)	Model	Detection speed (ms)	Couting accuracy(%)
**Zhang et al., (2021)**	80.3	/	stage	Faster-RCNN with Inception	169	/
**Zhou et al.(2019)**	86.8	0.874	88.3	Improved R-FCN	489	/
**Hayat et al. (2020)**	/	0.9028	89.77	Bayesian Segmentation	/	/
**Xu et al., (2020b)**	/	/	/	MHW-PD	/	87%
**Ours method**	90.32	0.89	82.36	YOLOV4-MobileNetV2-CBAM	22.5	85.37%
